# RANKL/RANK/OPG Pathway: A Mechanism Involved in Exercise-Induced Bone Remodeling

**DOI:** 10.1155/2020/6910312

**Published:** 2020-02-19

**Authors:** Mohammad Tobeiha, Mohammed H. Moghadasian, Negin Amin, Sadegh Jafarnejad

**Affiliations:** ^1^Research Center for Biochemistry and Nutrition in Metabolic Diseases, Kashan University of Medical Sciences, Kashan, Iran; ^2^Department of Food and Human Nutritional Sciences and the Canadian Centre for Agri-Food Research in Health and Medicine, University of Manitoba, Winnipeg, Canada

## Abstract

Bones as an alive organ consist of about 70% mineral and 30% organic component. About 200 million people are suffering from osteopenia and osteoporosis around the world. There are multiple ways of protecting bone from endogenous and exogenous risk factors. Planned physical activity is another useful way for protecting bone health. It has been investigated that arranged exercise would effectively regulate bone metabolism. Until now, a number of systems have discovered how exercise could help bone health. Previous studies reported different mechanisms of the effect of exercise on bone health by modulation of bone remodeling. However, the regulation of RANKL/RANK/OPG pathway in exercise and physical performance as one of the most important remodeling systems is not considered comprehensive in previous evidence. Therefore, the aim of this review is to clarify exercise influence on bone modeling and remodeling, with a concentration on its role in regulating RANKL/RANK/OPG pathway.

## 1. Introduction

Bones as an alive organ consist of about 70% mineral and 30% organic material. Calcium and phosphorous crystals, hydroxyapatite, and some ions such as sodium, fluoride, and magnesium are constituents of the mineral part. The organic part contains mostly collagen fiber and, in a lower amount, glycoproteins and proteoglycans [1]. The skeleton has several roles in the body such as protecting internal organs, frame of the body, and safe storage for some vital minerals like calcium. In contrast with what it seems, bones are a vivid tissue which is in turnover all the time [2]. About 200 million people are suffering from osteopenia and osteoporosis around the world; approximately 1 out of 3 women and 1 out of 5 men older than 50 years old have some forms of bone abnormalities [3]. From the population aging, it has been estimated that the prevalence of bone diseases would rise up in the near future. In the United States of America, it is estimated that bone disorders would increase 2.4 times in women and 3.1 times in men until 2050 [4].

Bones have various types of cells including osteoclasts, osteoblasts, osteocytes, and bone lining cells [5, 6]. Osteoblasts are originated from hematopoietic stem cells (HSCs, macrophage lineage of hematopoietic stem cells), and osteoclasts are originated from mesenchymal stem cells (MSCs) via some stages such as osteoprogenitors and preosteoblasts [7]. Fundamentally, bone modeling and remodeling include osteoclasts function in the removal of the bone surface and osteoblasts function on precipitating new matrix in them [8, 9]. This process is responsible for protecting skeleton function and fracture restoring. Any kind of defect in bone turnover coordination would result in bone diseases such as Paget's disease, fibrous dysplasia, osteoarthritis, osteoporosis, and fragility fractures [10–13].

Osteoclasts are the major cells in charge of bone resorption. They are positioned on the surface of bones and form trenches by their function. Activated osteoclasts release proteolytic enzymes which destroy connective tissues in bones. They also secrete some acids that resolve the mineral part of bones [14]. Through the different stages of osteoblasts differentiation, the level of some biomarkers, which are known as osteogenic markers, changes significantly. Among these markers, osteocalcin (OCL), Runx2, alkaline phosphatase, and osterix (Osx) can be named. On the other hand, for modulating monocyte-to-osteoclast differentiation, osteoblasts would release osteoprotegerin (OPG) and receptor activator of NF-kB ligand (RANKL), as well as macrophage colony-stimulating factor (M-SCF) [7, 15, 16]. RANKL/RANK, Wnt/b-catenin, and Jagged1/Notch1 are 3 important pathways modulated by osteoblasts which affect the bone mass density via the regulation of osteoblasts and osteoclasts functions [8]. In the RANKL/RANK/OPG pathway, RANKL binds to RANK as its receptor and eventually leads to osteoclast precursor maturation. Osteoprotegerin is known as a decoy receptor for RANKL which prevents RANKL-RANK binding and the following reactions [17].

There are several risk factors for bone health such as aging [18], estrogen deficiency, inflammation [14], metabolic diseases, improper diets [19], kidney dysfunction [20], side effects of some drugs like glucocorticoids [21], and oxidative stress [22]. There are various ways to protect the skeleton from disease and resorption or at least delay the onset of such disorders. For example, physical activity, healthy diets, and medical intervention can help the prevention of age-related bone loss or osteoporosis [18]. Several medications like bone resorption inhibitors and bone formation stimulators are in a postmenopausal treatment lineup [23]. These include bisphosphonates (e.g., alendronate) [24], strontium ranelate [25], denosumab (RANKL inhibitor) [26], and PTH [27]. A limitation in this kind of treatment is the risks of complications such as fever or muscle pain [28, 29]. Having a proper regimen that is nutrient-dense is one of the major strategies in saving and augmenting bone mass. Vitamin D, calcium, phosphorus, magnesium, zinc, and copper are some examples of necessary nutrients for skeleton health [4, 30, 31].

Planned physical activity is another useful plan for maintaining optimal bone health. It has been suggested that planned exercise would effectively regulate bone metabolism [29, 32]. Some studies have reported that exercise may postpone the beginning of osteoporosis by improving peak bone mass within adolescence [33, 34]. The exact mechanism by which exercise improves bone health is not clear yet. However, it has been accepted that increasing muscle mass and mechanical stress in bones results in boosting osteoblasts activities [35–37]. Previous studies reported different mechanisms covering the effect of exercise on bone health by modulation of bone remodeling. However, the regulation of RANKL/RANK/OPG pathway as one of the most important remodeling systems is not considered comprehensive in previous reports. Therefore, the aim of this review was to clarify exercise influence on bone modeling and remodeling, with a concentration on the role of the RANKL/RANK/OPG pathway.

## 2. The RANK/RANKL/OPG Pathway

The RANKL/RANK/OPG system is known for its roles in osteoclasts maturation, bone modeling, and bone remodeling. Receptor activator of NF-kB (RANK), receptor activator of NF-kB ligand (RANKL), and osteoprotegerin (OPG) are the main components of this signaling system. Interestingly, taking part in bone hemostasis is not the only effect of the RANKL/RANK/OPG pathway [2].

RANKL (also known as OPGL, ODF, and TRANCE), as a homotrimeric protein, is produced by osteoblasts and some other cells like activated T cells [38–40]. The secreted type of RANKL is a result of proteolytic division or alternative splicing on the membrane form [41]. Matrix metalloproteases (MMP3 or 7) and ADAM (a disintegrin and metalloprotease domain) are responsible for RANKL proteolytic cleavage [42, 43]. RANKL, which is a secretion of preosteoblasts, osteoblasts, osteocytes, and periosteal cells [44–46], make RANK activated, which is expressed by osteoclasts and its precursors [47]. RANKL has assignments for stimulating preosteoclasts' differentiation [48], adherence osteoclasts to bone tissue [49] and their following activation [48, 50], and their maintenance [51]. Preosteoclasts combine together and make a multinuclear cell which is affected by RANKL [8] not clear.

RANKL can be also produced by other organs such as thymus, lymph nodes, lung, and mammary glands, as well as the spleen and bone marrow [40]. RANKL might be released from epithelial cells in the lobules of mammary glands during pregnancy. Based on an animal study, RANKL helps in hyperplasia of these epithelial cells which is necessary for lactation and milk production [52].

RANK is also a homotrimeric transmembrane receptor from the TNF family. Its primary expression is limited to OPCs, dendritic cells, and mature osteoclasts [38]. RANK does not have innate protein kinase activating activity as other TNF family receptors have. All of the TRAFs 2, 5, and 6 bind to RANK but only TRAF 6 is required for bone health [53–56]. Aside from bone cells, RANK would be expressed by some carcinoma cells, such as breast cancer or prostate cancer, and also expressed in the mammary gland [52, 57, 58]. One of the roles of RANK that has received attention is its role in cancer cell proliferation; this makes RANK interesting in future therapy for cancers [57].

In addition to osteoblasts, there are plenty of cells that could express osteoprotegerins, such as the heart, liver, spleen, and kidney. A recent study suggested that B cells are in charge of 64% of bone marrow OPG expression [59]. As a TNF superfamily, OPG plays an anti-osteoclastogenesis role with binding to RANKL [60]. OPG takes part as a decoy receptor for RANKL and inhibiting RANKL-RANK binding through it. In fact, several agents that induce RANKL influence OPG regulation [61, 62]. Recent studies have shown that increases in plasma OPG levels in postmenopausal women lead to bone mass reinforcement [50]. Furthermore, in an experiment conducted by the use of mice, OPG was found to be a protector of large vessels from calcification [49]. Moreover, OPG has been suggested as an inhibitor of atherosclerotic plaque calcification [63].

## 3. RANKL/RANK/OPG Pathway and Bone Metabolism

Before the discovery of the RANKL/RANK/OPG signaling pathway in the 1990s, it was suggested that some agents expressed by osteoblasts are responsible for osteoclasts activation. But it was unexpected that these agents have been members of the TNF superfamily and they could have more functions than the bone turnover in the body [2]. Obviously, it is osteoblasts' task to recruit osteoclasts for bone resorption sites. Also, osteoblasts could regulate bone resorption by secreting OPG and RANKL. In fact, RANKL embedded from osteoclasts binds to its receptor (RANK) on the surface of OCPs and increases osteoclasts differentiation and mature osteoclasts. OPG could bind to RANKL and inhibits osteoclasts differentiation which means upregulation of OPG/RANKL ratio preventing osteoclastogenesis [64, 65]. Similar to other TNF family receptors, RANK does not have any innate protein kinase activities to regulate the signaling pathway. TRAF 6 is the only essential TRAF, among all TRAFs, that bind to RANK for regulating OCPs and osteoclasts activities. To support this claim, several studies have reported that a deficiency in TRAF 6 results in the development of osteoporosis [8, 53–55].

Probably the conclusive determinant in bone resorption is the RANKL/OPG ratio. Most of the time, both RANKL upregulation and OPG downregulation lead to bone loss [66]. There are several endogenous factors that affect the control of the RANKL/RANK/OPG system including some cytokines (TNF-a, IL-1, IL-6, IL-4, IL-11, and IL-17), hormones (vitamin D, estrogen, and glucocorticoids), and mesenchymal transcription factors [13, 67]. OPG is regulated by not only cytokines, hormones, and growth factors but also by Wnt/b-catenin [8, 68–70]. For osteoclast precursors conversion to mature osteoclasts, c-Fos is needed which is an activated transcription factor for RANKL [2, 71]. sRANKL is the soluble form of RANKL which appears in plasma. Elimination of RANKL and RANK in animal studies shows a major effect in inhibiting bone mass loss and osteoporosis [72]. Based on clinical observations, enhancing OPG concentrations in plasma leads to bone mass density augmentation in postmenopausal women [73].

In so many skeleton and nonskeletal disorders, alterations in RANKL and OPG proteins and their mRNA are observable [66]. Enhancement in ROS (reactive oxygen species) production through the function of NADPH oxidase enzymes controls osteoclastogenesis via regulating RANKL expression [14, 74]. Also, proinflammatory cytokines which increase in inflammatory conditions lead to the overexpression of RANKL by T cells that correlate with lower bone mass density (BMD) [56, 75]. In some pathological conditions like postmenopausal osteoporosis or arthritis rheumatoid which influenced hormones and cytokines level, bone resorption would significantly increase. These types of diseases would increase bone remodeling mostly through RANKL and M-CSF expression enhancement [2, 9].

Juvenile Paget disease is diagnosed via osteopenia, fractures, fast remodeling in woven bones, and development of bone deformities. In two Paget disease cases, depletion in OPG has been reported [38, 76]. Idiopathic hyperphosphatasia is also an autosomal osteopathic medicine and alterations in OPG level have a vital role in this disease. In this regard, OPG inactivation has been observed in some clinical trials [38, 69]. RANK signaling pathway is involved in a giant cell tumor of bone (GCTB) which is a rare and painful cancer. This cascade leads to excessive bone resorption and metastasis in these patients [77]. In rheumatoid arthritis, the inflammation advent results in overexpression in RANKL and subsequently bone-weakening [78].

### 3.1. Exercise and Bone Health

Exercise or planned physical activity is supposed to contribute to maintaining optimal health and healthy body weight [79, 80]. Exercise could indicate a “rejuvenating effect” and possibly prevent age-related skeleton disorders and bone resorption [18, 81]. Exercise has several advantages in protecting body health particularly bone modeling and remodeling [80]. The ability for bones to adjust with mechanical force and stress has been observed in the late 19^th^ century [82].

Mechanical load is one of the most important agents for bone mass density enhancement. The Mechanostat theory, which is first mentioned by Frost, expresses that bones have their own innate biological system to induce bone formation, in response to mechanical forces. This system includes bone cells, major osteocytes which are impressed by mechanical strain, transmitting it to the osteoclasts and osteoblasts and resulting in regulation of the skeleton homeostasis [37, 83, 84]. It has been accepted that mechanical forces help promote bone mass and strength. Interestingly, the skeleton could discriminate between internal force and strain-driven [85]. Osteoblasts, osteoclasts, and other bone cells are influenced by various endogen and exogen factors like cytokines. Proinflammatory and anti-inflammatory cytokines have major roles in skeleton modeling and remodeling [39]. Studies have shown that joint disorders like arthritis could make asymmetry pro- and anti-inflammatory cytokines, leading to bone loss [39, 86]. Exercise might increase anti-inflammatory cytokines and cause improvement in inflammatory cytokines [87, 88]. Also, mechanical load as an exercise regulates collagen synthesis during bone formation [89]. Muscle tension is transferred to the bones and leads to provoking osteoblasts proliferation [90]. In contrast, the lake of exercise, weightlessness, or bedridden would reduce osteoblasts activity and increase osteoclasts function [91].

Exercise is categorized into 6 classes: static weight-bearing exercises such as single-leg standing, high-impact weight-bearing exercises like running or dancing, low-impact weight-bearing exercises such as Tai Chi, high-impact non-weight-bearing exercises, low-impact non-weight-bearing exercises like swimming, and combination exercises [37, 92]. Investigations have indicated that regular physical activity with long duration and moderate intensity would decrease bone resorption and increase bone mass in both healthy and pathologic subjects [80]. Bone health would improve through weight-bearing exercises and helps bone density in growth, promoting bone health in aging [93–95]. In a 12-month clinical trial, postmenopausal inactive women with high doses of exercise had a greater bone mass density compared to women with moderate-dose exercise. This effect remained for almost a year after finishing study [29]. Intensive weight-bearing exercise may propagate P1NP, BAP, OPG, phosphate, and PTH levels [65, 96].

Exercise brings a cycle of reactions in the hypothalamus-hypophysis-adrenal line or hypothalamus-hypophysis-gonad line. These reactions stimulate some hormone expressions which help MSC differentiation to osteoblasts, including growth hormone, PTH, PGE2, and thyroid hormones [65, 97–100]. Sclerostin, a major role in bone formation, is a protein expressed by osteocytes. In fact, sclerostin supports bone mass by prohibiting the Wnt/B-catenin pathway. Wnt is a signaling pathway that proliferates osteoprogenitor and minimizes mature osteoblasts apoptosis. Exercise and the followed mechanical load lead to the reduction in bone sclerostin synthesis. Afterward, osteoblastic bone formation increases and osteoclastic bone loss decreases [37, 101]. There is some strong evidence that demonstrated exercise decline mRNA levels of markers from bone resorption like TRAP, cathepsin-K, and calcitonin receptors [102]. Moreover, participating in exercise could increase some osteogenic markers like OCL, Runx2, Osx, BAP, BMP2, and collagen type 1 in osteoblasts [15, 103–105]. It has been demonstrated that BAP and OCL, which are bone formation markers, are upregulated and TRAP (tartrate-resistant acid phosphatase), which is a bone resorption marker, is downregulated within an 8-week exercise plan in women [65, 103].

It has been shown that exercise promotes bone health through RANKL/RANK/OPG pathway regulation too [65].

### 3.2. Favorable Effects of Exercise on Bone Health by RANKL/RANK/OPG Regulation

**Figure 1:**
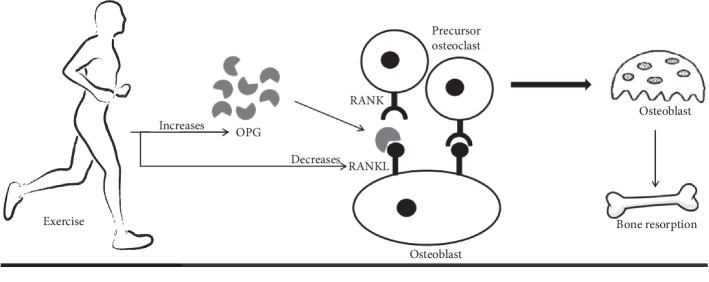
Interaction of exercise and RANKL/RANK/OPG biomolecular pathway. OPG: osteoprotegerin; RANK: receptor activator of nuclear factor *κ*B; RANKL: receptor activator of NF-kB ligand.

Figure 1 presents the details of the positive role of exercise in bone remodeling. Osteoblasts and osteoclasts are responsible for bone formation and bone resorption, respectively. So the effects of exercise on these 2 types of cells would help understand the association between exercise, bone modeling, and bone remodeling [88]. Exercise is responsible for the suppression in osteoclastogenesis and bone remodeling, which is mediated through the OPG/RANKL pathway released by osteoblasts, osteocytes, and MSCs [65].

There are multiple animal studies investigating the effect of chronic exercises on the pathway. In a study conducted using rats with CKD, the expression of RANKL and osteocalcin increased after endurance treadmill exercise [106]. Another study demonstrated the effects of exercise on glucocorticoid-induced osteoporotic that was investigated in rats. The results of this study confirmed that RANKL and RANKL-induced bone loss would be inhibited by vibration and treadmill training [107]. It was suggested that the treadmill and vibration stimulation exercise leads to a decrease in RANKL expression and an increase in OPG expression in the glucocorticoid-induced osteoporotic rats [107]. OPG and RANKL were meaningfully increased in response to 5-minute physical activity. In this study, which was performed on prednisolone-induced osteoporotic rats cells, treadmill and vibration platform training were used as examples of physical training. The results of the group treated with treadmill and vibration stimulation training indicated a subsequent decrease in RANKL and an increase in OPG levels [107]. Some limited animal studies reported beneficial effects of acute exercise on the pathway. Decreased RANKL levels and increased OPG levels have been observed in an experiment using acute training murine MC3T3-E1 osteoblasts [108]. In a chronic exercise study which was conducted in rats, an enhancement in the OPG/RANKL ratio was shown because of a decrease in RANKL expression [104]. On the other hand, an in vitro study has illustrated that mechanical strain could lead to abundance in OPG levels and decreases in M-CSF levels without alterations in RANKL levels in human osteoblasts [109]. Rubin et al. suggested that mechanical load could cause a reduction in RANKL, resulting in strong protection of bone loss and osteoclast proliferation [110].

Several previous studies reported the influence of acute exercises on the RANKL/RANK/OPG pathway. Scott et al. reported that acute endurance exercise causes an increase in levels of BAP and OPG in healthy men [96]. High-intensive acute exercise would enhance OPG and RANKL instantly. Also, this study has shown that 5-minute exercise increases IL-1a, IL-1B, IL-6, and TNF-a and 1-hour exercise brought them back to the basic levels [88]. The findings of another study suggested that endurance running made a reduction in sRANKL and an increase in OPG concentrations. The intensity in these results depends on the distance and duration of the path [111]. After a performance period with 80% VO2max and 40% VO2max intensity, OPG levels in serum have increased just under high-intensity exercise conditions in elderly women [112]. Based on a clinical trial done by Mezil et al., the low-impact high-intensity exercise would increase OPG, RANKL, and ALP levels in male university students [88]. The authors noted that, in the absence of adequate exercise, RANKL/RANK/OPG pathway effects would be minimal [113]. Acute training exercise with 60% or 80% VO2max intensity could not change serum RANKL and OPG levels, nor RANKL/RANK/OPG mRNA expression in college women [114].

In addition to acute exercise, the chronic exercises exert similar effects on RANKL/RANK/OPG pathway. In an investigation, long-term and intensive chronic exercise causes upregulation of OPG expression in postmenopausal women as compared to that in sedentary cases [115]. An article reported that after a 10-week high-impact walking plan, it significantly diminished RANKL levels without significant changes in OPG levels in middle-aged men [116]. Some findings suggested that RANKL and OPG levels and their expressions do not necessarily change by exercise. For example, a 32-week resistance exercise accompanied by weight-bearing exercise had no effect on RANKL and OPG levels and their ratio [117]. Likewise, in older women, after 8 months of resistance exercise or aerobic exercise, no significant changes were observed in RANKL and OPG levels [118]. After 12 weeks of combined exercise, Kim et al. found no significant changes in serum OPG and RANKL concentrations, nor in RANKL/RANK/OPG signaling mRNA expression [113].

Along with animal and human adult studies, there are several trails that include children and adolescents. These studies mostly focused on acute exercises. In one of these studies, the osteokines responses to rest and plyometric exercises in children have evaluated. Boys and girls with 10 years old on average were included in this study and the amounts of RANKL and OPG were measured before and after exercise (5 min, 1 hour, and 24 hours). In the pre-exercise analysis, it turned out that boys have higher levels of RANKL which indicated the discrimination in bone turnover between the two genders through the growth time. Girls showed a reduction in RANKL through exercise and it has kept reducing more when they continued to exercise till 24 h. OPG is enhanced by exercise; this enhancement is higher in boys specifically in 5 min and 1-hour exercise against girls which indicated the increment only on 24 h level of exercise [119]. The other survey has measured the plyometric exercise (high-impact) effects on bones in young females and the outcomes expressed a reduction in RANKL levels after 5-minute exercise. It stayed lower than the basic level (pre-exercise) until the end of 24 h exercise. However, OPG did not change at significant levels [120]. The finding of an investigation demonstrated that one session of plyometric exercise could increase both OPG and ALP (alkaline phosphatase) in boys and young men [121]. Another study investigated the bone mass impacts of exercise on adolescent girls. Participants were divided into 4 groups: high-impact exercise, medium-impact exercise, no-impact exercise, and leisure physical activity. The results represent no significant variation in OPG levels among groups; we have a slight reduction in OPG through growing just in a high-impact group. Also, RANKL levels increased along with age except in no-impact exercise (swimmers) which made a reduction in RANKL [122]. The comparison between the professional young female trainers who exercise 12–30 hours per week and nonathlete girls who do unplanned physical activity less than 3 hours in a week has shown that RANKL increases simultaneously with aging in both groups. No significant change was reported in them [123].  Table 1 displays the characteristics of the studies investigating the effects of different kinds of exercises on the RANKL/RANK/OPG pathway.

Despite strong evidence for the impact of mechanical loads on the RANKL/RANK/OPG signaling system, our knowledge is still limited on how this pathway may contribute to optimal bone health.

## 4. Conclusion

Based on the different studies that we reviewed, antithesis results have appeared. In most of the studies, exercise and physical activities promote bone health by increasing OPG and decreasing RANKL levels. However, there are several investigations that reported no change in OPG and RANKL levels after exercise. Interestingly, most of the experiments that we investigated have been carried out with high-intensity exercise. According to these studies, the actual effect of exercise on the RANKL/RANK/OPG system needs more investigations. Regardless, the positive impact of exercises on bone health and the overall well-being is undeniable.

## Figures and Tables

**Table 1:** General characteristics of the studies investigating the effects of exercises on RANKL/RANK/OPG regulation. ALP: alkaline phosphatase; BAP: bone alkaline phosphatase, OPG: osteoprotegerin; RANK: receptor activator of nuclear factor *κ*B; RANKL: receptor activator of NF-kB ligand.

Study name, year	Exercise type	Treatment time	Species/population/condition	Significant outcome
Scott et al. 2011 [96]	Acute, weight-bearing endurance exercise	8 days	Healthy men	OPG↑, BAP↑

Kish et al. 2015 [121]	Plyometric exercise	5 minutes, 1 hour, and finally 24 hours after exercise	Healthy boys and men	OPG↑, ALP↑

Bergström et al. 2011 [115]	Physical training (fast walking + aerobic training)	1 year	Postmenopausal women	OPG↑, RANKL↔, sclerostin↔

Rubin et al. 2000 [110]	Mechanical strain by a flexcell bioflex instrument	3 days	Murine bone stromal cells	RANKL↓

Notomi et al. 2014 [104]	Resistance training	8 weeks	Male Sprague Dawley rats	RANKL↓, OPG↔, OPG/RANKL↑

Mezil et al. 2015 [88]	High-intensity low-impact exercise	5 minutes after exercise	Male university students	ALP↑, OPG↑, RANKL↑
		1 hour after exercise		ALP↑
		24 hours after exercise		ALP↑

Troib et al. 2016 [106]	Endurance treadmill exercise	4 weeks	Young and growth-retarded chronic kidney disease rats	RANKL↑, Osteocalcin↑

Pichler et al. 2013 [107]	Treadmill and vibration stimulation training	NS	Osteoporosis rats	OPG↑, RANKL↓

Esen 2009 [116]	High-intensity walking (*n* = 14)	10 weeks	Middle-aged men	OPG↔, sRANKL↓

Esen 2009 [116]	Moderate-intensity walking (*n* = 13)	10 weeks	Middle-aged men	OPG↔, sRANKL↔

Ziegler et al. 2005 [111]	Endurance running distance of 42.195 km	The first 30 minutes of finishing the run	Long-distance runners	sRANKL↓, OPG↑

Ziegler et al. 2005 [111]	Endurance running shorter distance of 15.8 km	The first 30 minutes of finishing the run	Long-distance runners	sRANKL↓, OPG↔

Tang et al. 2006 [108]	Cyclic tensile strain using a flexercell strain unit with 6%, 12% or 18% elongation	24 hours	Murine MC3T3-E1 osteoblasts	OPG↑, OPG mRNA expression↑, sRANKL↓, RANKL mRNA expression↓ (magnitude-dependent)

Kim et al. 2019 [113]	Combined exercise	12 weeks	Healthy college females	OPG↔, RANKL↔, RANKL/RANK/OPG signaling mRNA expression↔

Saunders et al. 2006 [109]	Small-scale loading machine that imparts via bending	3 hours	Osteoblastic MG-63 cells	OPG↑, RANKL↔, OPG/RANK ratio↑

Kim et al. 2017 [112]	Acute exercise of high (80% VO2max) intensity	Immediately after and then recovery 60 minutes after exercise	Osteopenia elderly women	OPG↑, RANKL↔

Kim et al. 2017 [112]	Acute exercise of low (40% VO2max) intensity	Immediately after and then recovery 60 minutes after exercise	Osteopenia elderly women	OPG↔, RANKL↔

Marques et al. 2013 [117]	Resistance exercise accompanied by weight-bearing exercise	32 weeks	Healthy older adults	RANKL↔, OPG↔, OPG/RANKL ratio↔

Marques et al. 2011 [118]	Resistance exercise (RE)	8 months	Older women	RANKL↔, OPG↔, OPG/RANKL ratio↔

Marques et al. 2011 [118]	Aerobic exercise (AE)	8 months	Older women	RANKL↔, OPG↔, OPG/RANKL ratio↔

Kim et al. 2018 [114]	Acute exercise of high (80% VO2max) intensity	Immediately after and then recovery 90 minutes after exercise	Healthy college females	RANKL↔, OPG↔, RANKL/RANK/OPG pathway mRNA expression↔

Kim et al. 2018 [114]	Acute exercise of moderate (60% VO2max) intensity	Immediately after and then recovery 90 minutes after exercise	Healthy college females	RANKL↔, OPG↔, RANKL/RANK/OPG pathway mRNA expression↔

Klentrou et al. 2018 [119]	Rest and following plyometric exercise (5 min, 1 h, and 24 h)	24 hours	Boys and girls (10 years old in average)	Girls: OPG↑, RANKL↓ Boys: OPG↑, RANKL↑

Dekker et al. 2017 [120]	1 resting and 3 after exercise (5 min, 1 h, and 24 h)	24 hours	Premenarcheal and postmenarcheal girls	RANKL↓OPG↔ OPG/RANKL↑

Maïmoun et al. 2011 [123]	Training 12–30 h/week) professional athlete (compared with free-time physical activity ≤ 3 h/week (nonathlete)	—	Girls (age 10–17.2 years)	OPG↔RANKL↑

Maïmoun et al. 2013 [122]	Participants are divided into 4 groups: high-impact exercise, medium-impact exercise, no-impact exercise, and leisure physical activity	—	Girls from 10.7 to 18.0 years old	OPG↔RANKL↑

## References

[1] Vannucci L. (2018). Calcium intake in bone health: a focus on calcium-rich mineral waters. *Nutrients*.

[2] Boyce B. F., Xing L. (2007). Biology of RANK, RANKL, and osteoprotegerin. *Arthritis Research & Therapy*.

[3] Cooper C., Campion G., Melton L. J. (1992). Hip fractures in the elderly: a world-wide projection. *Osteoporosis International*.

[4] Seem S. A., Yuan Y. V., Tou J. C. (2019). Chocolate and chocolate constituents influence bone health and osteoporosis risk. *Nutrition*.

[5] Datta H. K., Ng W. F., Walker J. A., Tuck S. P., Varanasi S. S. (2008). The cell biology of bone metabolism. *Journal of Clinical Pathology*.

[6] Florencio-Silva R., da Silva Sasso G. R., Sasso-Cerri E., Jesus Simões M., Sérgio Cerri P. (2015). Biology of bone tissue: structure, function, and factors that influence bone cells. *BioMed Research International*.

[7] Thompson W. R., Rubin C. T., Rubin J. (2012). Mechanical regulation of signaling pathways in bone. *Gene*.

[8] Boyce B. F., Xing L. (2008). Functions of RANKL/RANK/OPG in bone modeling and remodeling. *Archives of Biochemistry and Biophysics*.

[9] Boyce B. F., Xing L., Shakespeare W. (2003). Regulation of bone remodeling and emerging breakthrough drugs for osteoporosis and osteolytic bone metastases. *Kidney International*.

[10] Khosla S., Oursler M. J., Monroe D. G. (2012). Estrogen and the skeleton. *Trends in Endocrinology & Metabolism*.

[11] Adams D. J., Rowe D. W., Ackert-Bicknell C. L. (2016). Genetics of aging bone. *Mammalian Genome*.

[12] Sobacchi C., Schulz A., Coxon F. P., Villa A., Helfrich M. H. (2013). Osteopetrosis: genetics, treatment and new insights into osteoclast function. *Nature Reviews Endocrinology*.

[13] Nardone V., D’Asta F., Brandi M. (2014). Pharmacological management of osteogenesis. *Clinics*.

[14] Mundy G. R. (2007). Osteoporosis and inflammation. *Nutrition Reviews*.

[15] Chen D., Zhao M., Mundy G. R. (2004). Bone morphogenetic proteins. *Growth Factors*.

[16] Hu R., Liu W., Li H. (2011). A Runx2/miR-3960/miR-2861 regulatory feedback loop during mouse osteoblast differentiation. *Journal of Biological Chemistry*.

[17] Boyce B. F., Xing L. (2007). The RANKL/RANK/OPG pathway. *Current Osteoporosis Reports*.

[18] Santos L., Elliott-Sale K. J., Sale C. (2017). Exercise and bone health across the lifespan. *Biogerontology*.

[19] Willems H. M. E., van den Heuvel E. G. H. M., Schoemaker R. J. W., Klein-Nulend J., Bakker A. D. (2017). Diet and exercise: a match made in bone. *Current Osteoporosis Reports*.

[20] Covic A., Vervloet M., Massy Z. A. (2018). Bone and mineral disorders in chronic kidney disease: implications for cardiovascular health and ageing in the general population. *The Lancet Diabetes & Endocrinology*.

[21] Buckley L., Humphrey M. B. (2018). Glucocorticoid-induced osteoporosis. *New England Journal of Medicine*.

[22] Chen J.-R., Shankar K., Nagarajan S., Badger T. M., Ronis M. J. J. (2008). Protective effects of estradiol on ethanol-induced bone loss involve inhibition of reactive oxygen species generation in osteoblasts and downstream activation of the extracellular signal-regulated kinase/signal transducer and activator of transcription 3/receptor activator of nuclear factor-kappaB ligand signaling cascade. *Journal of Pharmacology and Experimental Therapeutics*.

[23] Brar K. (2010). Prevalent and emerging therapies for osteoporosis. *Medical Journal Armed Forces India*.

[24] Boivin G. Y., Chavassieux P. M., Santora A. C., Yates J., Meunier P. J. (2000). Alendronate increases bone strength by increasing the mean degree of mineralization of bone tissue in osteoporotic women. *Bone*.

[25] Marie P. J. (2007). Strontium ranelate: new insights into its dual mode of action. *Bone*.

[26] Hanley D. A., Adachi J. D., Bell A., Brown V. (2012). Denosumab: mechanism of action and clinical outcomes. *International Journal of Clinical Practice*.

[27] Pazianas M. (2015). Anabolic effects of PTH and the “anabolic window”. *Trends in Endocrinology & Metabolism*.

[28] Alami S. (2016). Barriers to effective postmenopausal osteoporosis treatment: a qualitative study of patients’ and practitioners’ views. *PLoS One*.

[29] Gonzalo-Encabo P., McNeil J., Boyne D. J., Courneya K. S., Friedenreich C. M. (2019). Dose-response effects of exercise on bone mineral density and content in postmenopausal women. *Scandinavian Journal of Medicine & Science in Sports*.

[30] Rizzoli R. (2014). Nutritional aspects of bone health. *Best Practice & Research Clinical Endocrinology & Metabolism*.

[31] Levis S., Lagari V. S. (2012). The role of diet in osteoporosis prevention and management. *Current Osteoporosis Reports*.

[32] Russo C. R. (2009). The effects of exercise on bone. Basic concepts and implications for the prevention of fractures. *Clinical Cases in Mineral and Bone Metabolism: The Official Journal of the Italian Society of Osteoporosis, Mineral Metabolism, and Skeletal Diseases*.

[33] Tveit M., Rosengren B. E., Nilsson J. Å., Karlsson M. K. (2015). Exercise in youth: high bone mass, large bone size, and low fracture risk in old age. *Scandinavian Journal of Medicine & Science in Sports*.

[34] Warden S. J., Fuchs R. K., Castillo A. B., Nelson I. R., Turner C. H. (2007). Exercise when young provides lifelong benefits to bone structure and strength. *Journal of Bone and Mineral Research : The Official Journal of the American Society for Bone and Mineral Research*.

[35] Fleg J. L. (2012). Aerobic exercise in the elderly: a key to successful aging. *Discovery Medicine*.

[36] Palombaro K. M., Black J. D., Buchbinder R., Jette D. U. (2013). Effectiveness of exercise for managing osteoporosis in women postmenopause. *Physical Therapy*.

[37] Hong A. R., Kim S. W. (2018). Effects of resistance exercise on bone health. *Endocrinology and Metabolism*.

[38] Wada T., Nakashima T., Hiroshi N., Penninger J. M. (2006). RANKL-RANK signaling in osteoclastogenesis and bone disease. *Trends in Molecular Medicine*.

[39] Takayanagi H. (2007). Osteoimmunology: shared mechanisms and crosstalk between the immune and bone systems. *Nature Reviews Immunology*.

[40] Kearns A. E., Khosla S., Kostenuik P. J. (2008). Receptor activator of nuclear factor kappaB ligand and osteoprotegerin regulation of bone remodeling in health and disease. *Endocrine Reviews*.

[41] Ikeda T., Kasai M., Utsuyama M., Hirokawa K. (2001). Determination of three isoforms of the receptor activator of nuclear factor-kappaB ligand and their differential expression in bone and thymus. *Endocrinology*.

[42] Lynch C. C., Hikosaka A., Acuff H. B. (2005). MMP-7 promotes prostate cancer-induced osteolysis via the solubilization of RANKL. *Cancer Cell*.

[43] Hikita A., Yana I., Wakeyama H. (2006). Negative regulation of osteoclastogenesis by ectodomain shedding of receptor activator of NF-kappaB ligand. *Journal of Biological Chemistry*.

[44] Collin-Osdoby P. (2004). Regulation of vascular calcification by osteoclast regulatory factors RANKL and osteoprotegerin. *Circulation Research*.

[45] Silvestrini G. (2005). Detection of osteoprotegerin (OPG) and its ligand (RANKL) mRNA and protein in femur and tibia of the rat. *Journal of Molecular Histology*.

[46] Nakashima T., Hayashi M., Fukunaga T. (2011). Evidence for osteocyte regulation of bone homeostasis through RANKL expression. *Nature Medicine*.

[47] Hsu H., Lacey D. L., Dunstan C. R. (1999). Tumor necrosis factor receptor family member RANK mediates osteoclast differentiation and activation induced by osteoprotegerin ligand. *Proceedings of the National Academy of Sciences*.

[48] Lacey D. L., Timms E., Tan H.-L. (1998). Osteoprotegerin ligand is a cytokine that regulates osteoclast differentiation and activation. *Cell*.

[49] Bucay N., Sarosi I., Dunstan C. R. (1998). osteoprotegerin-deficient mice develop early onset osteoporosis and arterial calcification. *Genes & Development*.

[50] Li J., Sarosi I., Yan X.-Q. (2000). RANK is the intrinsic hematopoietic cell surface receptor that controls osteoclastogenesis and regulation of bone mass and calcium metabolism. *Proceedings of the National Academy of Sciences*.

[51] Li J. (2001). Combination of TNF-alpha and IL-1 beta induced osteoclast formation and bone resorption is dependent on RANK signal transduction. *Journal of Bone and Mineral Research*.

[52] Fata J. E., Kong Y.-Y., Li J. (2000). The osteoclast differentiation factor osteoprotegerin-ligand is essential for mammary gland development. *Cell*.

[53] Kim H.-H., Lee D. E., Shin J. N. (1999). Receptor activator of NF-kappaB recruits multiple TRAF family adaptors and activates c-Jun N-terminal kinase. *FEBS Letters*.

[54] Lomaga M. A., Yeh W.-C., Sarosi I. (1999). TRAF6 deficiency results in osteopetrosis and defective interleukin-1, CD40, and LPS signaling. *Genes & Development*.

[55] Naito A., Azuma S., Tanaka S. (1999). Severe osteopetrosis, defective interleukin-1 signalling and lymph node organogenesis in TRAF6-deficient mice. *Genes to Cells*.

[56] Turk N., Cukovic-Cavka S., Korsic M., Turk Z., Vucelic B. (2009). Proinflammatory cytokines and receptor activator of nuclear factor kappaB-ligand/osteoprotegerin associated with bone deterioration in patients with crohn’s disease. *European Journal of Gastroenterology & Hepatology*.

[57] Kim N.-S., Kim H.-J., Koo B.-K. (2006). Receptor activator of NF-kappaB ligand regulates the proliferation of mammary epithelial cells via Id2. *Molecular and Cellular Biology*.

[58] Chen G., Sircar K., Aprikian A., Potti A., Goltzman D., Rabbani S. A. (2006). Expression of RANKL/RANK/OPG in primary and metastatic human prostate cancer as markers of disease stage and functional regulation. *Cancer*.

[59] Li Y., Toraldo G., Li A. (2007). B cells and T cells are critical for the preservation of bone homeostasis and attainment of peak bone mass in vivo. *Blood*.

[60] Lala R., Matarazzo P., Bertelloni S., Buzi F., Rigon F., de Sanctis C. (2000). Pamidronate treatment of bone fibrous dysplasia in nine children with McCune-Albright syndrome. *Acta Paediatrica (Oslo, Norway: 1992)*.

[61] Boyle W. J., Simonet W. S., Lacey D. L. (2003). Osteoclast differentiation and activation. *Nature*.

[62] Hofbauer L. C., Schoppet M. (2004). Clinical implications of the osteoprotegerin/RANKL/RANK system for bone and vascular diseases. *JAMA*.

[63] Morony S., Tintut Y., Zhang Z. (2008). Osteoprotegerin inhibits vascular calcification without affecting atherosclerosis in ldlr (-/-) mice. *Circulation*.

[64] Ominsky M. S., Li X., Asuncion F. J. (2008). RANKL inhibition with osteoprotegerin increases bone strength by improving cortical and trabecular bone architecture in ovariectomized rats. *Journal of Bone and Mineral Research*.

[65] Yuan Y., Chen X., Zhang L. (2016). The roles of exercise in bone remodeling and in prevention and treatment of osteoporosis. *Progress in Biophysics and Molecular Biology*.

[66] Kostenuik P. (2005). Osteoprotegerin and RANKL regulate bone resorption, density, geometry and strength. *Current Opinion in Pharmacology*.

[67] Hofbauer L. C., Heufelder A. E. (2001). Role of receptor activator of nuclear factor-kappaB ligand and osteoprotegerin in bone cell biology. *Journal of Molecular Medicine*.

[68] Theoleyre S., Wittrant Y., Tat S. K., Fortun Y., Redini F., Heymann D. (2004). The molecular triad OPG/RANK/RANKL: involvement in the orchestration of pathophysiological bone remodeling. *Cytokine & Growth Factor Reviews*.

[69] Cundy T. (2002). A mutation in the gene TNFRSF11B encoding osteoprotegerin causes an idiopathic hyperphosphatasia phenotype. *Human Molecular Genetics*.

[70] Glass D. A., Bialek P., Ahn J. D. (2005). Canonical Wnt signaling in differentiated osteoblasts controls osteoclast differentiation. *Developmental Cell*.

[71] Karsenty G., Wagner E. F. (2002). Reaching a genetic and molecular understanding of skeletal development. *Developmental Cell*.

[72] Simonet W. S., Lacey D. L., Dunstan C. R. (1997). Osteoprotegerin: a novel secreted protein involved in the regulation of bone density. *Cell*.

[73] Samelson E. J., Broe K. E., Demissie S. (2008). Increased plasma osteoprotegerin concentrations are associated with indices of bone strength of the hip. *The Journal of Clinical Endocrinology & Metabolism*.

[74] Weaver C. M., Alekel D. L., Ward W. E., Ronis M. J. (2012). Flavonoid intake and bone health. *Journal of Nutrition in Gerontology and Geriatrics*.

[75] Sylvester F. A., Wyzga N., Hyams J. S. (2007). Natural history of bone metabolism and bone mineral density in children with inflammatory bowel disease. *Inflammatory Bowel Diseases*.

[76] Whyte M. P., Obrecht S. E., Finnegan P. M. (2002). Osteoprotegerin deficiency and juvenile paget’s disease. *New England Journal of Medicine*.

[77] Wu P.-F., Tang J.-Y., Li K.-H. (2015). RANK pathway in giant cell tumor of bone: pathogenesis and therapeutic aspects. *Tumor Biology*.

[78] Geusens P. (2012). The role of RANK ligand/osteoprotegerin in rheumatoid arthritis. *Therapeutic Advances in Musculoskeletal Disease*.

[79] Neufer P. D., Bamman M. M., Muoio D. M. (2015). Understanding the cellular and molecular mechanisms of physical activity-induced health benefits. *Cell Metabolism*.

[80] Qi Z., Liu W., Lu J. (2016). The mechanisms underlying the beneficial effects of exercise on bone remodeling: roles of bone-derived cytokines and microRNAs. *Progress in Biophysics and Molecular Biology*.

[81] Loprinzi P. D., Loenneke J. P., Blackburn E. H. (2015). Movement-based behaviors and leukocyte telomere length among US adults. *Medicine & Science in Sports & Exercise*.

[82] Burgers T. A., Williams B. O. (2013). Regulation of Wnt/beta-catenin signaling within and from osteocytes. *Bone*.

[83] Frost H. M. (2003). Bone’s mechanostat: a 2003 update. *The Anatomical Record*.

[84] Klein-Nulend J., Bakker A. D., Bacabac R. G., Vatsa A., Weinbaum S. (2013). Mechanosensation and transduction in osteocytes. *Bone*.

[85] Nagasawa S., Honda A., Sogo N., Umemura Y. (2008). Effects of low-repetition jump exercise on osteogenic response in rats. *Journal of Bone and Mineral Metabolism*.

[86] Schett G. (2011). Effects of inflammatory and anti-inflammatory cytokines on the bone. *European Journal of Clinical Investigation*.

[87] Ullum H., Haahr P. M., Diamant M. (1994). Bicycle exercise enhances plasma IL-6 but does not change IL-1 alpha, IL-1 beta, IL-6, or TNF-alpha pre-mRNA in BMNC. *Journal of Applied Physiology*.

[88] Mezil Y. A., Allison D., Kish K. (2015). Response of bone turnover markers and cytokines to high-intensity low-impact exercise. *Medicine & Science in Sports & Exercise*.

[89] Huiskes R., Ruimerman R., van Lenthe G. H., Janssen J. D. (2000). Effects of mechanical forces on maintenance and adaptation of form in trabecular bone. *Nature*.

[90] Kaspar D., Seidl W., Neidlinger-Wilke C., Beck A., Claes L., Ignatius A. (2002). Proliferation of human-derived osteoblast-like cells depends on the cycle number and frequency of uniaxial strain. *Journal of Biomechanics*.

[91] Meyers V. E., Zayzafoon M., Douglas J. T., McDonald J. M. (2005). RhoA and cytoskeletal disruption mediate reduced osteoblastogenesis and enhanced adipogenesis of human mesenchymal stem cells in modeled microgravity. *Journal of Bone and Mineral Research*.

[92] Howe T. E., Shea B., Dawson L. J. (2011(7)). Exercise for preventing and treating osteoporosis in postmenopausal women. *Cochrane Database of Systematic Reviews*.

[93] Specker B., Thiex N. W., Sudhagoni R. G. (2015). Does exercise influence pediatric bone? A systematic review. *Clinical Orthopaedics and Related Research*.

[94] Zhao R., Zhang M., Zhang Q. (2017). The effectiveness of combined exercise interventions for preventing postmenopausal bone loss: a systematic review and meta-analysis. *Journal of Orthopaedic & Sports Physical Therapy*.

[95] Gil-Diaz M. C., Raynor J., O’Brien K. O., Schwartz G. J., Weber D. R. (2019). Systematic review: associations of calcium intake, vitamin D intake, and physical activity with skeletal outcomes in people with type 1 diabetes mellitus. *Acta Diabetologica*.

[96] Scott J. P., Sale C., Greeves J. P., Casey A., Dutton J., Fraser W. D. (2011). The role of exercise intensity in the bone metabolic response to an acute bout of weight-bearing exercise. *Journal of Applied Physiology*.

[97] Pomerants T. (2008). Impact of acute exercise on bone turnover and growth hormone/insulin-like growth factor axis in boys. *Journal of Sports Medicine and Physical Fitness*.

[98] Remes T., Väisänen S. B., Mahonen A. (2004). The association of bone metabolism with bone mineral density, serum sex hormone concentrations, and regular exercise in middle-aged men. *Bone*.

[99] Boeloni J. N., Ocarino N. M., Melo A. B. (2009). Dose-dependent effects of triiodothyronine on the osteogenic differentiation of rat bone marrow mesenchymal stem cells. *Hormone Research*.

[100] Shamir D., Keila S., Weinreb M. (2004). A selective EP4 receptor antagonist abrogates the stimulation of osteoblast recruitment from bone marrow stromal cells by prostaglandin E2 in vivo and in vitro. *Bone*.

[101] Galea G. L., Lanyon L. E., Price J. S. (2017). Sclerostin’s role in bone’s adaptive response to mechanical loading. *Bone*.

[102] Suzuki N., Yoshimura Y., Deyama Y., Suzuki K., Kitagawa Y. (2008). Mechanical stress directly suppresses osteoclast differentiation in RAW264.7 cells. *International Journal of Molecular Medicine*.

[103] Lester M. E., Urso M. L., Evans R. K. (2009). Influence of exercise mode and osteogenic index on bone biomarker responses during short-term physical training. *Bone*.

[104] Notomi T., Karasaki I., Okazaki Y. (2014). Insulinogenic sucrose+amino acid mixture ingestion immediately after resistance exercise has an anabolic effect on bone compared with non-insulinogenic fructose+amino acid mixture in growing rats. *Bone*.

[105] Wang Q. S. (2015). A comparative study of mechanical strain, icariin and combination stimulations on improving osteoinductive potential via NF-kappaB activation in osteoblast-like cells. *Biomed Eng Online*.

[106] Troib A., Guterman M., Rabkin R., Landau D., Segev Y. (2016). Endurance exercise and growth hormone improve bone formation in young and growth-retarded chronic kidney disease rats. *Nephrology Dialysis Transplantation*.

[107] Pichler K., Loreto C., Leonardi R., Reuber T., Weinberg A. M., Musumeci G. (2013). RANKL is downregulated in bone cells by physical activity (treadmill and vibration stimulation training) in rat with glucocorticoid-induced osteoporosis. *Histology and Histopathology*.

[108] Tang L., Lin Z., Li Y.-M. (2006). Effects of different magnitudes of mechanical strain on osteoblasts in vitro. *Biochemical and Biophysical Research Communications*.

[109] Saunders M. M., Taylor A. F., Du C., Zhou Z., Pellegrini V. D., Donahue H. J. (2006). Mechanical stimulation effects on functional end effectors in osteoblastic MG-63 cells. *Journal of Biomechanics*.

[110] Rubin J., Murphy T., Nanes M. S., Fan X. (2000). Mechanical strain inhibits expression of osteoclast differentiation factor by murine stromal cells. *American Journal of Physiology-Cell Physiology*.

[111] Ziegler S., Niessner A., Richter B. (2005). Endurance running acutely raises plasma osteoprotegerin and lowers plasma receptor activator of nuclear factor *κ* B ligand. *Metabolism*.

[112] Kim C.-S., Kim H.-J., Kim J.-Y. (2017). The effects of exercise intensity difference on bone metabolic markers and cytokines of the RANKL/RANK/OPG system in Korean osteopenia elderly women. *Exercise Science*.

[113] Kim J.-Y., Kim H.-J., Kim C.-S. (2019). Effects of 12-week combined exercise on RANKL/RANK/OPG signaling and bone-resorption cytokines in healthy college females. *Journal of Exercise Nutrition & Biochemistry*.

[114] Kim J.-Y., Kim H.-J., Park D.-H., Shin Y.-A., Min S.-K., Kim C.-S. (2018). The effects of acute exercise on RANKL/RANK/OPG pathway and bone metabolic markers in healthy college female. *Exercise Science*.

[115] Bergström I., Parini P., Gustafsson S. A., Andersson G., Brinck J. (2011). Physical training increases osteoprotegerin in postmenopausal women. *Journal of Bone and Mineral Metabolism*.

[116] Esen H. (2009). Do walking programs affect C-reactive protein, osteoprotegerin and soluble receptor activator of nuclear factor-kappa*β* ligand?. *Turkish Journal of Biochemistry*.

[117] Marques E. A., Mota J., Viana J. L. (2013). Response of bone mineral density, inflammatory cytokines, and biochemical bone markers to a 32-week combined loading exercise programme in older men and women. *Archives of Gerontology and Geriatrics*.

[118] Marques E. A., Wanderley F., Machado L. (2011). Effects of resistance and aerobic exercise on physical function, bone mineral density, OPG and RANKL in older women. *Experimental Gerontology*.

[119] Klentrou P., Angrish K., Awadia N., Kurgan N., Kouvelioti R., Falk B. (2018). Wnt signaling-related osteokines at rest and following plyometric exercise in prepubertal and early pubertal boys and girls. *Pediatric Exercise Science*.

[120] Dekker J., Nelson K., Kurgan N., Falk B., Josse A., Klentrou P. (2017). Wnt signaling-related osteokines and transforming growth factors before and after a single bout of plyometric exercise in child and adolescent females. *Pediatric Exercise Science*.

[121] Kish K., Mezil Y., Ward W. E., Klentrou P., Falk B. (2015). Effects of plyometric exercise session on markers of bone turnover in boys and young men. *European Journal of Applied Physiology*.

[122] Maïmoun L., Coste O., Philibert P. (2013). Peripubertal female athletes in high-impact sports show improved bone mass acquisition and bone geometry. *Metabolism*.

[123] Maïmoun L., Coste O., Mariano-Goulart D. (2011). In peripubertal girls, artistic gymnastics improves areal bone mineral density and femoral bone geometry without affecting serum OPG/RANKL levels. *Osteoporosis International*.

